# Genome-wide analysis identified novel susceptible genes of restless legs syndrome in migraineurs

**DOI:** 10.1186/s10194-022-01409-9

**Published:** 2022-03-29

**Authors:** Yun-Jin Jiang, Cathy Shen-Jang Fann, Jong-Ling Fuh, Ming-Yi Chung, Hui-Ying Huang, Kuo-Chang Chu, Yen-Feng Wang, Chia-Lin Hsu, Lung-Sen Kao, Shih-Pin Chen, Shuu-Jiun Wang

**Affiliations:** 1 Institute of Molecular and Genomic Medicine, National Health Research Institutes, Miaoli County, 35053 Taiwan; 2 Biotechnology Center, National Chung Hsing University, Taichung, Taiwan; 3 Institute of Biomedical Sciences, Academia Sinica, Taipei, 11529 Taiwan; 4 Department of Neurology, Neurological Institute, Taipei Veterans General Hospital, Taipei, 11217 Taiwan; 5 School of Medicine, College of Medicine, National Yang Ming Chiao Tung University, Taipei, 11221 Taiwan; 6 Department of Life Sciences & Institute of Genome Sciences, National Yang Ming Chiao Tung University, Taipei, 11221 Taiwan; 7 Department of Medical Research, Taipei Veterans General Hospital, Taipei, 11217 Taiwan; 8 Brain Research Center, National Yang Ming Chiao Tung University, Taipei, 11221 Taiwan; 9 Institute of Clinical Medicine, National Yang Ming Chiao Tung University, Taipei, 11221 Taiwan

**Keywords:** Migraine, Restless legs syndrome, Genome-wide association study, Zebrafish, *VSTM2L* and *CCDC141*

## Abstract

**Background:**

Restless legs syndrome is a highly prevalent comorbidity of migraine; however, its genetic contributions remain unclear.

**Objectives:**

To identify the genetic variants of restless legs syndrome in migraineurs and to investigate their potential pathogenic roles.

**Methods:**

We conducted a two-stage genome-wide association study (GWAS) to identify susceptible genes for restless legs syndrome in 1,647 patients with migraine, including 264 with and 1,383 without restless legs syndrome, and also validated the association of lead variants in normal controls unaffected with restless legs syndrome (*n* = 1,053). We used morpholino translational knockdown (morphants), CRISPR/dCas9 transcriptional knockdown, transient CRISPR/Cas9 knockout (crispants) and gene rescue in one-cell stage embryos of zebrafish to study the function of the identified genes.

**Results:**

We identified two novel susceptibility loci rs6021854 (in *VSTM2L*) and rs79823654 (in *CCDC141*) to be associated with restless legs syndrome in migraineurs, which remained significant when compared to normal controls. Two different morpholinos targeting *vstm2l* and *ccdc141* in zebrafish demonstrated behavioural and cytochemical phenotypes relevant to restless legs syndrome, including hyperkinetic movements of pectoral fins and decreased number in dopaminergic amacrine cells. These phenotypes could be partially reversed with gene rescue, suggesting the specificity of translational knockdown. Transcriptional CRISPR/dCas9 knockdown and transient CRISPR/Cas9 knockout of *vstm2l* and *ccdc141* replicated the findings observed in translationally knocked-down morphants.

**Conclusions:**

Our GWAS and functional analysis suggest *VSTM2L* and *CCDC141* are highly relevant to the pathogenesis of restless legs syndrome in migraineurs.

**Supplementary Information:**

The online version contains supplementary material available at 10.1186/s10194-022-01409-9.

## Background

Migraine is a highly prevalent and disabling neurological disorder, which is comorbid with a variety of neuropsychiatric disorders, including an intriguing sensorimotor disease-restless legs syndrome (RLS) [1, 2]. Restless legs syndrome (RLS) is an intriguing sensorimotor disorder characterized by an urge to move legs, which occurs mostly at night and disturbs sleep, being exacerbated by lying down with unpleasant sensations in legs, and can be temporarily relieved by voluntary leg movements [3]. Evidence has suggested complex associations between migraine and RLS. The prevalence of RLS in patients with migraine [1] could be up to seven times higher than that in the general population [4]. The severity of RLS in patients with migraine is worse than that of non-migraineurs [5], and the occurrence of RLS is more frequent in chronic compared with episodic migraineurs [6]. Moreover, RLS and migraine were found to have bidirectional trigger effects [7]. Yet, detailed mechanisms underlying comorbid RLS in migraineurs are unclear.

Both migraine and RLS are known to have high heritability, and genome-wide association studies (GWASs) have made substantial progress in identifying susceptibility genes for both diseases [8–17]. Dysfunctional dopaminergic neurotransmission and iron homeostasis have been proposed to be common mechanisms shared by RLS [18–20] and migraine [21, 22]; however, the genetic constituents contributing to RLS in migraineurs remain to be explored. We previously identified that a single-nucleotide polymorphism (SNP) rs2300478 at *MEIS1,* the gene responsible for iron homeostasis [23], increased the risk of RLS by 1.42-fold in migraine subjects via a candidate gene approach [24]. A recent small-scaled GWAS also suggested additional genes may contribute to RLS in migraineurs [25]. To further decipher the role of genetic variants in RLS in patients with migraine, we implemented a two-stage GWAS followed by in vivo functional analyses with zebrafish [26–28].

## Methods

### Study participants and data collection

A two-stage case–control GWAS was implemented to identify susceptible genes for RLS in migraineurs by comparing the cases (i.e., migraineurs with RLS) with controls (i.e., migraineurs without RLS). The significant findings of the discovery cohort were validated in the replication cohort, and a combined analysis of both cohorts was employed to examine the significance of the validated SNPs. In addition, we also examined the significant SNPs in an independent normal control cohort unaffected with restless legs syndrome or migraine. Consecutive patients with migraine were enrolled in the headache clinic of Taipei Veterans General Hospital (TVGH). They filled out a structured questionnaire with questions regarding personal information, medical history, and headache history. Participants were interviewed and their questionnaires and medical records were reviewed simultaneously by board-certified neurologists specialized in headache diagnosis. Migraine was diagnosed according to the criteria proposed in the International Classification of Headache Disorders, 3^rd^ edition [29]. Subjects with secondary headache disorders except for medication overuse headache were excluded. RLS was diagnosed based on the criteria proposed by the International RLS Study Group [30]. Subjects with ferritin < 50 ng/ml, anaemia, creatinine > 1.5 mg/dL or pregnancy were eliminated to exclude secondary RLS. Subjects with any RLS symptom proposed in the criteria or periodic limb movements in sleep based on self-reported nocturnal leg jerks during sleep were excluded from the control groups.

### Genotyping in the discovery cohort

We genotyped 642,832 SNPs using the Affymetrix Axiom Genome-Wide CHB 1 Array Plate, which has high coverage of genome-wide common variants for Han Chinese. SNP genotypes were called using the Axiom GT1 algorithm. Quality control (QC) criteria were applied to exclude SNPs if they (a) were monomorphic in both cases and controls, (b) had a total call rate of less than 95%, (c) had a minor allele frequency of less than 5% and a total call rate of less than 99%, or (d) showed significant (*P* < 1 × 10^–8^) deviation from Hardy–Weinberg equilibrium in controls. For sample filtering, arrays with generated genotypes for < 95% of the loci were excluded.

Heterozygosity of SNPs on the X-chromosome was used to verify the sex of the samples. PLINK version 1.09 [31] was used to identify samples with genetic relatedness, indicating that they were from the same individual (or monozygotic twins) or from first-, second- or third-degree relatives. These determinations were made based on evidence for cryptic relatedness from identity-by-descent status (pi-hat cut-off of 0.125).

### Genotyping in the replication cohorts

We selected SNPs that were within 200 kb of a gene which contains at least two adjacent SNPs with a *P* value of < 1 × 10^–4^. Single SNPs with a trend *P* value < 1 × 10^–4^ but not within 200 kb of a gene were not chosen for replication because we aimed to explore known protein coding genes. Genotyping was performed in replication cohorts using the Sequenom MassARRAY iPLEX platform (Sequenom Inc., San Diego, CA, USA). Genotyping in both cohorts are services provided by the National Center for Genome Medicine (NCGM).

### Imputation for the discovery case–control GWAS

We conducted a genotype imputation analysis in the discovery cohort using the 1000 Genomes Phase 3 reference data by implementing IMPUTE2 [32]. Well-imputed SNPs (info score > 0.4) were retained followed by systematic QC as described above.

### Morpholino translational knockdown

Morpholino oligonucleotide can block translation by targeting the 5’ untranslated region (UTR) of mRNA or inhibit RNA splicing by targeting exon/intron junctions. We designed six 25-base morpholinos (Gene Tools, Philomath, OR) that target the 5’UTR or splicing junction of *ccdc141* and *vstm2l* (Additional file 1).

### CRISPR interference

CRISPR gRNAs were designed with Benchling and the cloning sequences are shown in Additional file 2. Oligonucleotides were annealed in a thermoblock at 95 °C for 5 min and cooled to room temperature. Annealed oligonucleotides were cloned into pT7-gRNA plasmid at BsmBI site and verified by sequencing. To make dCas9 mRNA, dead Cas9 plasmid [33] was linearized by XbaI enzyme and purified by Gel extraction kit (Qiagen, Hilden, Germany). mRNA was synthesized by mMESSAGE mMACHINE T3 kit (Life Technologies, Carlsbad, CA) and purified by RNeasy mini kit (Qiagen). To make gRNA mRNA, pT7-gRNA plasmid was linearized by BamHI enzyme and purified by Gel extraction kit. RNA probe was synthesized by in vitro transcription using a MEGAscript® T7 Transcription kit (Thermo Fisher Scientific, Waltham, MA) and purified by ethanol precipitation.

### Transient and stable CRISPR/Cas9 knockout (KO)

The CRISPR/Cas9 KO is carried out by a non-for-profit service offered by the Taiwan Zebrafish Technology and Resource Center (TZTRC) according to previous reports. Briefly, together with the common tracrRNA and Cas9 protein, 4 gene-specific crRNAs (Additional file 3; Horizon, Waterbeach, UK), 2 for each gene, were injected into one-cell stage embryos separately [34]. Transient CRISPR/Cas9-injected embryos (crispants) have been demonstrated to largely phenocopy mutants [35]. The CRISPR/Cas9 activity detection and mutation screening were performed by high resolution melting analysis [36]. The stable KOs were confirmed by Sanger sequencing and maintained according to the standard operating protocol [37].

### Tyrosine hydroxylase RNA in situ hybridization

Tyrosine hydroxylase (TH) is an enzyme responsible for the biosynthesis of dopamine precursors. The 3–5 dpf wild-type and injected embryos were used for in situ hybridization following previously established protocol [38]. The embryos were fixed in 4% fresh-made paraformaldehyde at 4 °C overnight and then treated with 3% H_2_O_2_ and 5% KOH for depigmentation. Embryos were washed and transferred into 100% methanol at -20 °C overnight. Digoxigenin-labelled antisense RNA probes were used for labelling to detect the distribution of dopaminergic cells, and then the embryos were mounted in glycerol for observation and photography.

### Fin movement observation

We utilized a video system under normal laboratory lighting to observe pectoral fin movement and evaluate whether the injected embryos had hyperkinetic movements mimicking the “restlessness” and “urge to move the limbs” in patients with RLS. The 5 dpf embryos were used because pectoral fins and body organs are relatively well-developed. Embryos were mounted on glass slides covered with 1% low melting agar and put under a dissecting microscope to observe fin movements. One-to-three-minute videos were filmed by DFK 23UP031 USB Camera (The Imaging Source Asia Co., Taipei, Taiwan). Video Analysis Tools, After Effects and Tracker (Adobe, San Jose, CA), were used. The average flapping frequency (times/second) was acquired by catching the fin movement in *x* and time in *y* coordinates.

### Quantitative RT-PCR (qRT-PCR)

Dechorionated 2 dpf embryos were collected and total RNA was extracted by RNAzol® RT reagent (Molecular Research Center, Inc.). cDNA was synthesized by SuperScript™ III Reverse Transcriptase kit (Thermo Fisher Scientific). The experiment was conducted by LightCycler® 480 Instrument II with SensiFAST™ SYBR® Hi-ROX kit (Bioline). *Actin* was used as an internal control in all triplicated experiments. The qPCR data was analysed by LightCycler® 480 software version 1.5.0.39.

### Statistics

Association analyses were carried out by comparing allele/genotype frequencies between cases and controls using a single-point method: Cochran–Armitage trend test. The distribution of expected *P* values under the null hypothesis and genomic inflation value (λ) were calculated. The Manhattan and quantile–quantile (Q-Q) plots were created using the R package [39]. Genetic analyses were conducted using PLINK (version 1.09) [31]. Detection of possible population stratification was carried out by using principal component analysis (PCA) implemented in EIGENSTRAT to infer continuous axes of genetic variation. We adjusted for potential genetic heterogeneity by incorporating the first 10 PCs in the logistic regression tests of association with RLS. Joint analysis was conducted by combining data from the discovery and replication samples. In addition, we also examined the association of significant variants with migraine in an independent migraine case–control cohort. For studies involving zebrafish, data are reported as the mean ± SD or median and interquartile range. Student’s t test was used for comparison of continuous variables; Mann–Whitney U test was used for comparisons of unpaired nonparametric variables. All calculated *P*-values were two-tailed, and statistical significance was defined as *P*-value less than 0.05. These analyses were performed using Graphpad Prism, version 7.00 (GraphPad Software, La Jolla, CA).

## Results

### Association analysis

**Fig. 1:**
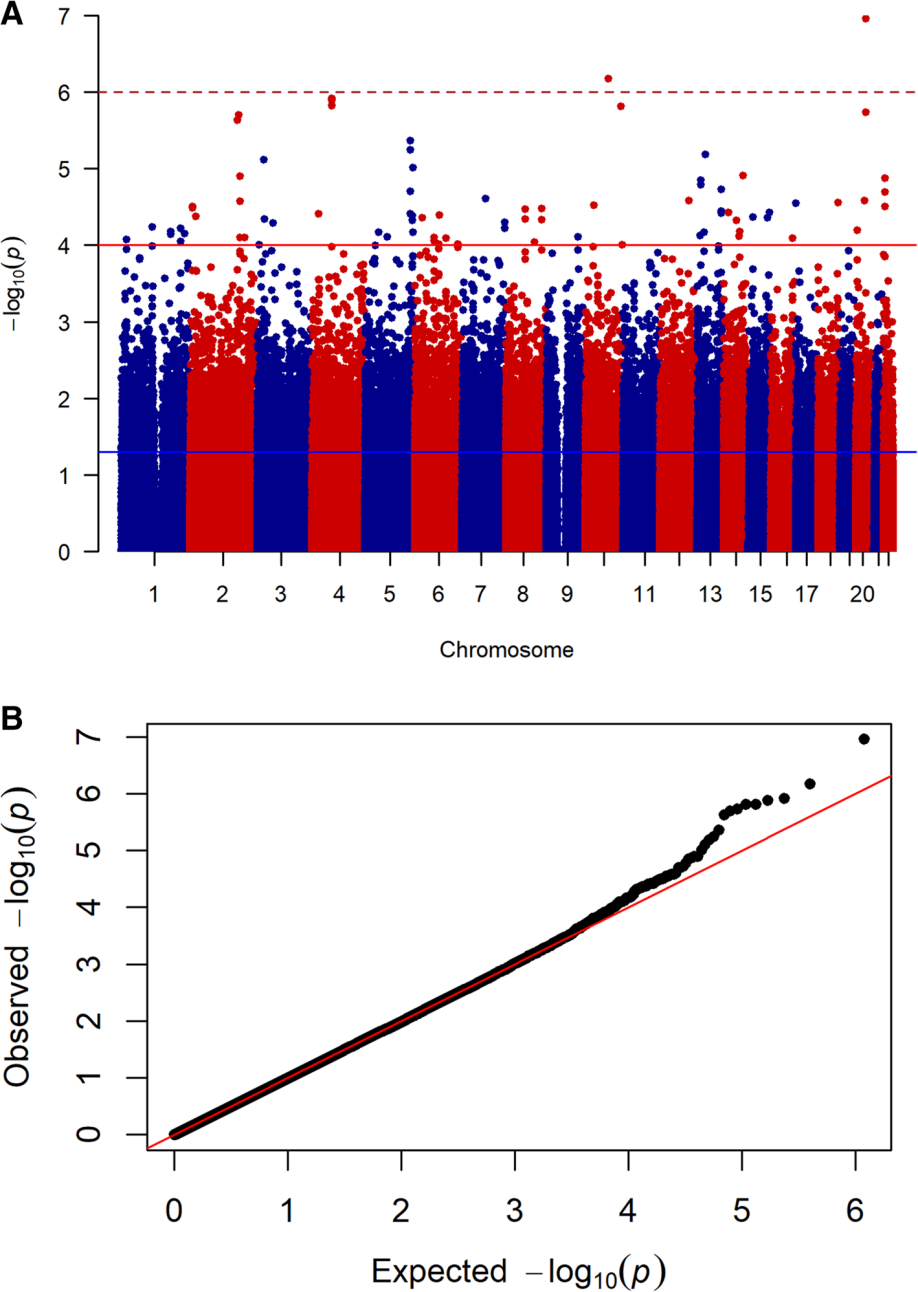
**A** Manhattan plot for RLS association in patients with migraine. Manhattan plot of the discovery genome-wide association analysis of 115 cases and 635 controls. The *x* axis is chromosomal position, and the *y* axis is the significance (–log_10_ *P*) of association derived from Cochran–Armitage trend tests. **B** Quantile–quantile plot of results from the Cochran-Mantel–Haenszel analysis. Red line represents the distribution of *P* values under the null hypothesis, given a study inflation factor (λ) of 1.000

Demographic characteristics of participants including age and sex were not significantly different between cases (migraineurs with RLS) and controls (migraineurs without RLS) in the discovery (age: 38.7 ± 12.4 vs. 39.0 ± 12.5 yrs, *P* = 0.775; female: 87.0% vs. 78.9%, *P* = 0.056) or replication cohort (age: 40.4 ± 12.6 vs. 39.5 ± 11.9 yrs, *P* = 0.378; female: 82.6% vs. 76.6%, *P* = 0.132). In the discovery stage, we genotyped 115 migraine patients with RLS and 635 migraine patients without RLS using the Affymetrix Axiom Genome-Wide CHB 1 Array Plate (Fig. 1A). After applying stringent QC criteria, we obtained 590,468 (91.85%) SNPs with an average call rate of 99.6 ± 0.5%. The value of the genomic inflation factor was 1.000, suggesting that there was no evidence for population stratification (Fig. 1B). PCA based on genotype data from 590,468 SNPs with equal spacing across the human genome showed no outliers. In total, 81 SNPs showed significant (*p* < 10^–4^) association signals with RLS. Four of the significant SNPs within or near (within 200 kb) genes were genotyped, and an additional 3 SNPs in the region were included for fine mapping in the replication cohort consisting of 149 migraine patients with RLS and 748 migraine patients without RLS (Additional file 4).

### rs79823654 in CCDC141 and rs6021854 in VSTM2L are associated with risk of RLS in migraineurs

**Fig. 2:**
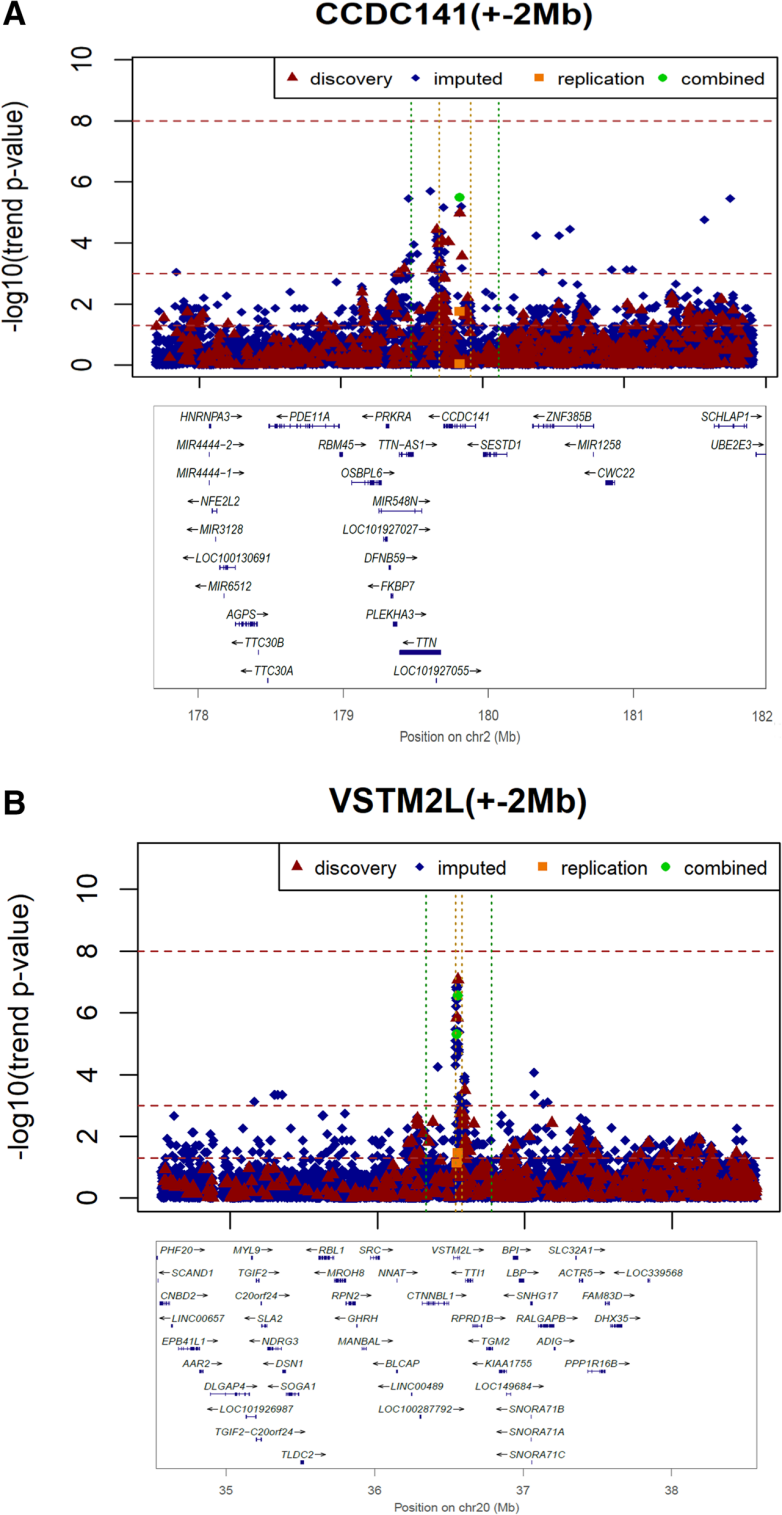
Regional plots of association signals. Regional plots for two newly identified loci associated with risk of restless legs syndrome in patients with migraine. Each regional plot shows the chromosomal position (GRCh37/hg19) of SNPs in the specific region against –log_10_ *P* values from association results of genotyped and imputed SNPs in stage 1 GWAS samples and stage 2 replication samples

We identified two novel loci: rs79823654 in *CCDC141* and rs6021854 in *VSTM2L* that were significantly associated with the risk of RLS in migraineurs in both discovery and replication cohorts (Table 1, Fig. 2). In the discovery dataset, rs6021854 and rs79823654 were the most significant SNP, which remained significant after adjustment for PC1‒PC10 of population structure. The association between these two SNPs and RLS in migraineurs was further confirmed in the replication dataset with a similar genetic impact. Joint analysis of both cohorts demonstrated that both SNPs were associated with an increased risk of RLS in migraineurs (Table 1). By comparing these cases (i.e., migraineurs with RLS) with normal controls, these two variants remained significant (Table 1).

**Table 1:** Association results for restless legs syndrome in patients with migraine

SNP	Gene	Chr	Position	Risk allele	stage	RAF		OR (95%CI)		*P* value	The *P* value adjusted
						**Case**	**Control**			**(trend)**	
rs79823654	*CCDC141*	2	179,839,018	A	1	0.130	0.053	2.740	(1.715–4.377)	1.05 × 10^–5^	2.51 × 10^–5^
					2	0.101	0.061	1.642	(1.084–2.486)	0.017	0.0179
					Joint	0.113	0.057	2.046	(1.501–2.788)	3.27 × 10^–6^	5.81 × 10^–6^
					R1	0.113	0.066	1.857	(1.344–2.565)	2.75 × 10^–4^	1.76 × 10^–4^
rs6021854	*VSTM2L*	20	36,545,927	A	1	0.252	0.116	2.447	(1.738–3.446)	8.63 × 10^–8^	4.69 × 10^–7^
					2	0.182	0.136	1.421	(1.021–1.977)	0.036	0.03598
					Joint	0.213	0.127	1.838	(1.451–2.328)	2.73 × 10^–7^	4.63 × 10^–7^
					R1	0.213	0.154	1.504	(1.175–1.925)	9.73 × 10^–4^	1.19 × 10^–3^

*SNP* single nucleotide polymorphism, *Chr* chromosome, *OR* odds ratio for risk allele, *CI* confidence interval, *PC* principal component; Stage 1 (GWAS) included 115 cases and 635 controls; Stage 2 (replication stage) included 149 cases and 748 controls; Joint: Combining stage 1 and 2; R1: combined cases (264 migraineurs with RLS) vs. 1,053 normal controls

*P* value is derived from trend test, the *P* value adjusted is derived from the logistic regression adjusted with age and sex; Risk allele, allele with higher frequency in cases compared to controls. All genomic information is from human genome build hg19

### Association of RLS with SNPs within dopamine receptor or tyrosine hydroxylase genes

To gain insight on the potential association between dopamine and RLS, we also examined whether the SNPs in tyrosine hydroxylase *(TH)* or dopaminergic receptors (*DRD1*, *DRD2*, *DRD3*, *DRD4*, and *DRD5*) have different allele frequencies between patients with and without RLS. Among the 414 SNPs within these genes available in our imputation data, none have reached our pre-defined significance level (*p* < 1 × 10^–4^). Only 17 SNPs within *DRD1*, *DRD2* or *TH* have shown borderline significance (1 × 10^–4^ < *p* < 5 × 10^–4^) in association with RLS (see Additional file 5).

### Expression pattern of ccdc141 and vstm2l in zebrafish

Expression of *ccdc141* and *vstm2l* in 1–4 dpf embryos was shown in Additional file 6. Their expression patterns in zebrafish are similar to those of mice and rats [40, 41].

### Morpholino translational knockdown of ccdc141 and vstm2l

Six morpholinos targeting the 5’ UTR and splicing junction on *ccdc141* and *vstm2l* were injected into one-cell stage embryos. We checked the success rate of translational knockdown and compared the morphological and phenotypic differences between wild-type embryos and morphants (Additional file 7). Successful translational knockdown was observed in *ccdc141* 5’UTR (MO1) and *vstm2l* splicing (MO2) morphants, because the amacrine cell number can be restored by injecting *ccdc141* mRNA in the former (also see below) and splicing morpholino caused a pre-terminated *vstm2l* transcript in the latter (Additional file 7). These morphants were selected for further evaluation.

### Altered expression of th-positive cells in morphants

**Fig. 3:**
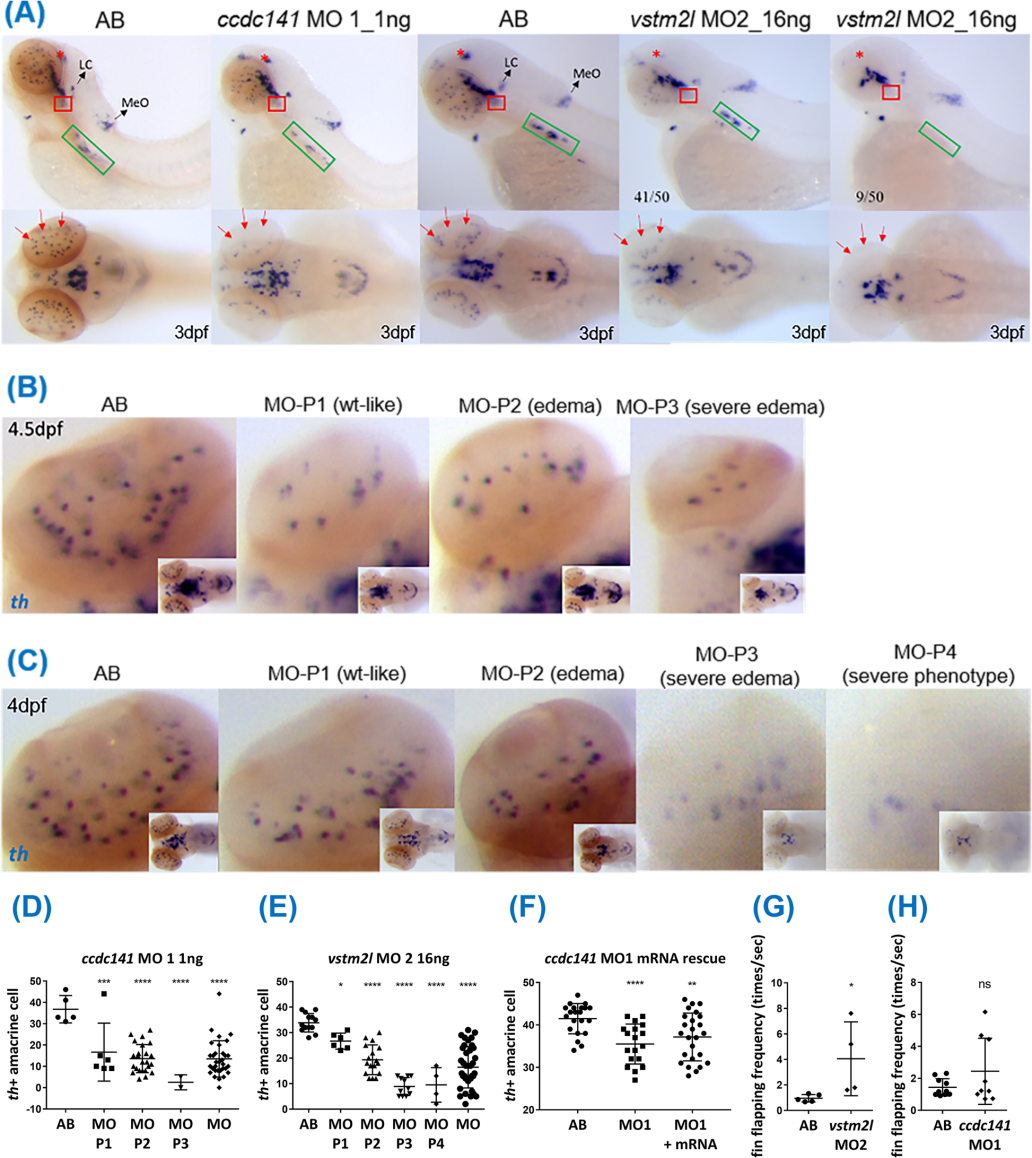
Expression of *tyrosine hydroxylase (th)* and fin movement frequency in *ccdc141* and *vstm2l* morphants. In situ hybridization was conducted with *tyrosine hydroxylase* antisense RNA probe. At 3 dpf, (**A**) in *ccdc141* 5’UTR (MO1) morphants, the distribution of *th* is dispersed and the *th*-expressing amacrine cells (red arrows) are decreased; in *vstm2l* splicing (MO2) morphants, lower *th* expression in dorsal pretectum (red asterisk), DC7 neurons (red square) and amacrine cells was observed; and the distribution of *th* in sympathetic superior cervical ganglion (SCG, green rectangle) is decreased and dispersed. Of note, the *th* expression in locus coeruleus (LC) and medulla oblongata (MeO) neurons does not alter. For quantification before *th *in situ experiments, the morphants were separated into groups according to their phenotypic severity. The results showed that fewer *th*-expressing amacrine cells were observed in every group, including wt-like (P1) group in (**B**) 4.5 dpf *ccdc141* MO1 morphants and (**C**) 4 dpf *vstm2l* MO2 morphants, whose statistical data are shown in (**D**) embryos injected with 1 ng *ccdc141* MO1 morpholino and (**E**) embryos injected with 16 ng *vstm2l* MO2 morpholino, respectively. Note that (**F**) amacrine cell deceasing phenotype was rescued by co-injecting *ccdc141* mRNA into *ccdc141* morphants. **G** Hyperkinetic movements were observed in *vstm2l* MO2 morphants with significant differences. **H** The *ccdc141* MO1 morphants had a trend of hyperkinetic movements, though the *P* value was not significant. (In these experiments, *ccdc141* MO1 morphants were injected with 0.5 ng *ccdc141* MO1 morpholino, and *vstm2l* MO2 morphants were injected with 16 ng *vstm2l* MO2 morpholino. Only wild-type like embryos were used to conduct experiments.) (N number for (**A**) AB = 2, *ccdc141* MO1_1ng = 7, AB = 4, *vstm2l* MO2_16ng = 41, *vstm2l* MO2_16ng_ severe phenotype = 9. (**D**) AB = 5, MO-P1 = 6, MO-P2 = 24, MO-P3 = 2, MO = 32. (**E**) AB = 13, MO-P1 = 6, MO-P2 = 17, MO-P3 = 11, MO-P4 = 4, MO = 38. (**F**) AB = 21, MO1 = 18, MO1 + mRNA = 25. **G** *vstm2l* AB = 5, *vstm2l* MO2 = 4. **H** *ccdc141* AB = 10, *ccdc141* MO1 = 9.) (Mann–Whitney U test was used for comparisons of unpaired nonparametric variables. All calculated *P*-values were two-tailed, and statistical significance was defined as *P*-value less than 0.05. Symbol meaning: *, *p* ≤ 0.05; **, *p* ≤ 0.01; ***, *p* ≤ 0.001; ****: *p* ≤ 0.0001)

Because the pathogenesis of RLS is considered to be associated with dopaminergic neurotransmission, we compared the distribution of *th*-positive cells (most of which are dopaminergic) in wild-type and morphant embryos (Fig. 3A). While pretectum, retinal amacrine cells, DC1-6 neurons, and DC7 neurons are dopaminergic, locus coeruleus (LC) and medulla oblongata (MeO) neurons are noradrenergic [42, 43]. However, sympathetic superior cervical ganglion (SCG) [44, 45] neurons are mainly adrenergic, with a few cells exhibiting a cholinergic phenotype [46, 47]. We found that in *ccdc141* 5’UTR (MO1) morphants, the distribution of *th* was dispersed and the *th*-expressing amacrine cells were decreased; in *vstm2l* splicing (MO2) morphants, lower *th* expression in pretectum, DC7 neurons and amacrine cells was observed. The distribution of *th* in SCG neurons was decreased and dispersed (Fig. 3A). In contrast, *th* expression in LC and MeO neurons did not change in both morphants (Fig. 3A). The morphants were further divided into groups according to their phenotypic severity before *th *in situ hybridization. We still found fewer *th*-expressing amacrine cells in all groups among *ccdc141* MO1 morphants (Fig. 3B and D) and *vstm2l* MO2 morphants (Fig. 3C and D). The decrease of amacrine cells was partially rescued by co-injecting *ccdc141* mRNA into *ccdc141* morphants (Fig. 3F), suggesting that the phenotype is specific.

### RLS-relevant behavioural phenotypes in morphants

We observed hyperkinetic movements of pectoral fins in 5 dpf *vstm2l* MO2 morphants (Fig. 3G) (see Additional file 8 for video), resembling the core phenotypes, restlessness and urge to move the limbs, of RLS. The *ccdc141* MO1 morphants also had a trend of hyperkinetic movements (Fig. 3H and Additional file 7).

### Transcriptional knockdown of ccdc141 and vstm2l recapitulates findings in morphants

We then performed transcriptional genetic knockdown of *ccdc141* and *vstm2l* by CRISPR interference (CRISPRi) [33]. Four CRISPRi gRNAs were designed for each gene. The gRNA was injected into one-cell stage embryos separately and its effect was measured by qPCR. The gRNAs of *ccdc141* gRNA1, gRNA3, gRNA4 and *vstm2l* gRNA3 that can repress the expression level of target gene down to approximately 0.5-fold examined by qRT-PCR were used to conduct the following experiments (Additional file 9). The embryos injected with *ccdc141* gRNA4 caused reduced *th*-positive amacrine cells and exhibited hyperkinetic movement compared with non-injected embryos (Additional file 10). The *ccdc141* gRNA1 and *vstm2l* gRNA3 only caused decreased *th*-positive amacrine cells (Additional files 9 and 10), suggesting the possibility of different genetic thresholds for different phenotypes.

### Transient knockout of ccdc141 and vstm2l recapitulates the findings in knocked-down embryos

**Fig. 4:**
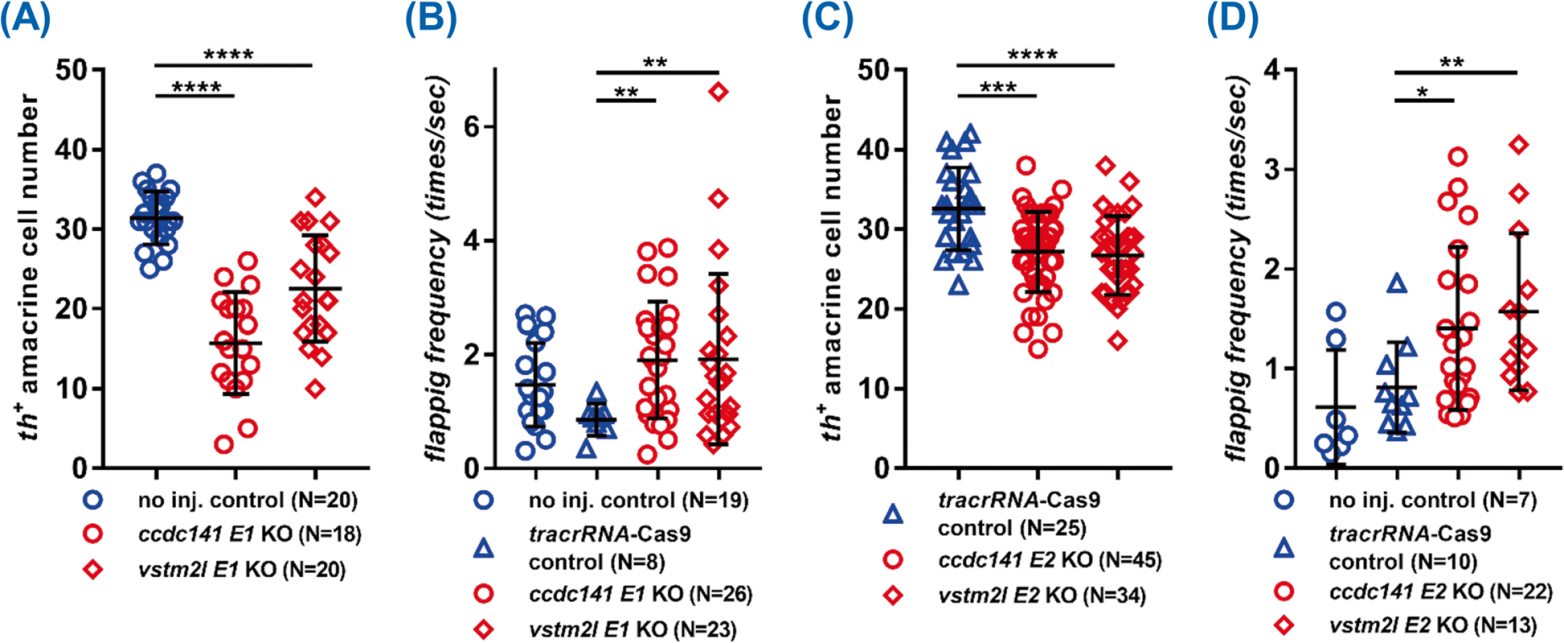
The quantity of amacrine cells and flapping frequency of pectoral fins in the transient *ccdc141* and *vstm2l* knocked-out embryos. The statistical data showed that fewer *th*-expressing amacrine cells were observed in 4 dpf (**A**) *ccdc141* and *vstm2l* exon 1 (E1) knocked-out embryos**.** A statistically significant hyperkinetic movements were found in 5 dpf (**B**) *ccdc141* and *vstm2l* exon 1 knocked-out embryos. Fewer *th*-expressing amacrine cells were observed with statistical data in 4 dpf (**C**) *ccdc141* and *vstm2l* exon 2 (E2) knocked-out embryos. Hyperkinetic movements were found statistically significant in 5 dpf (**D**) *ccdc141* and *vstm2l* exon 2 knocked-out embryos, when compared to tracrRNA-Cas9 control**.** The not-injected AB wildtype embryos were used as no inj. control; for the exon 1-targeting knockouts in (**A**) and (**B**), 200 pg *tracrRNA* and 200 pg Cas9 protein were injected per embryo as basic *tracrRNA*-Cas9; basic *tracrRNA*-Cas9 and 50 pg *ccdc141*-*crRNA* E1/*vstm2l*-*crRNA* E1 were injected per embryo for *ccdc141* exon 1*/vstm2l* exon 1 KO. For the exon 2-targeting knockouts in (**C**) and (**D**), 138 pg *tracrRNA* and 461 pg Cas9 protein were injected per embryo as basic *tracrRNA*-Cas9; basic *tracrRNA*-Cas9 and 34.6 pg *ccdc141*-*crRNA* E2/*vstm2l*-*crRNA* E2 were injected per embryo for *ccdc141* exon 2/*vstm2l* exon 2 KO. Mann–Whitney U test was used for comparisons of unpaired nonparametric variables. All calculated *P*-values were two-tailed, and statistical significance was defined as *P*-value less than 0.05. Symbol meaning: *, *p* ≤ 0.05; **, *p* ≤ 0.01; ***, *p* ≤ 0.001; ****, *p* ≤ 0.0001

Transient CRISPR KO cause phenotypes in crispants indistinguishable to those of loss-of-function mutants [35, 48]. We, therefore, further used CRISPR/Cas9 to transiently knock out *ccdc141* and *vstm2l* and aimed to generate stable KO lines. Two sets of crRNAs were used to target exons 1 and 2 of each gene (Additional file 3). In the exon 1 crispants of two genes, the number of *th*-expressing amacrine cells in the eyes is decreased (Fig. 4A) and the movement of pectoral fins is hyperkinetic (Fig. 4B). The exon 2 crispants had similar phenotypes (Fig. 4C and D). These results repeat the conclusion obtained from translational and transcriptional knockdowns.

We can only identify stable KO lines from the offspring of exon 2 CRISPR/Cas9-injected F0: three *ccdc141* lines and two *vstm2l* lines (Additional file 11). Unexpectedly, F2 embryos from two examined KO lines showed neither decreased *th*-expressing amacrine cells nor hyperkinetic fin movement (Additional file 12). Interestingly, the expression of corresponding gene in homozygotes is diminished (Additional file 12).

## Discussion

By using a two-stage GWAS, we identified two novel susceptibility genes, *VSTM2L* and *CCDC141*, accountable for an increased risk of RLS in patients with migraine. These two genes were highly expressed in the central nervous system (CNS) among species. Inhibiting expression of these two genes at the transcriptional or translational level resulted in morphological changes involving fin development, decreased number of dopaminergic neurons, and hyperkinetic movements of pectoral fins in zebrafish, compatible with the clinical symptoms and putative pathogenic pathways of RLS. Gene rescue reversed the phenotypes of the morphants, which further supports that these findings are not due to non-specific toxic effects from morpholino and augmented the functional roles of these two genes in RLS pathogenesis.

Our data confirmed the crucial role of *VSTM2L* and *CCDC141* in RLS in patients with migraine; however, pre-existing information regarding these two genes is scarce. *VSTM2L*, short for V-set and transmembrane domain containing 2 like, was previously known as C20orf102. The protein encoded by *VSTM2L* has an exquisitely CNS-specific expression and is known to be a secreted antagonist of a neuroprotective mitochondrial peptide Humanin [40]. *CCDC141* (short for coiled-coil domain containing 141), also named CAMDI after coiled-coil protein associated with myosin II and DISC1 (disrupted in schizophrenia 1), is known to affect neuronal development by impairing radial migration through DISC1 and myosin II-mediated centrosome positioning [41]. How these known functions of *VSTM2L* and *CCDC141* contribute to RLS is unclear, but our data indicate that it might be mediated through affecting the development and distribution of dopaminergic neurons. The A11 dopaminergic nucleus of the dorsal-posterior hypothalamus has been considered to be important in the pathogenesis of RLS [19] and migraine [21] in rodent models. In zebrafish, we also demonstrated that inhibition of the expression of *vstm2l* and *ccdc141* could affect the distribution of dopaminergic cells in the CNS. Though the *th* expression level of DC2,4–6 (A-11 type, the rodent A11 equivalent) [43] did not change, that of DC7, which is considered as caudal hypothalamus, did decline (Fig. 3A). Of note, the distribution of DC2,4–6 neurons seem dispersed in morphants. Nevertheless, we could not exclude the possibility that it was due to morphological changes. Interestingly, the *th* expression of A11-type dopaminergic neurons, LC and MeO neurons with far-ranging projections is not affected, while that of DC7 neurons and retinal amacrine cells projecting exclusively locally or to adjacent brain regions is decreased [42]. Evolutionarily, there is no direct zebrafish counterpart of mammalian substantia nigra/ventral tegmental area dopaminergic neurons. A trans-species comparison of the A11-type and other dopaminergic systems, which are also less well studied in mammals [43], and behavioral phenotypes need to be examined.

Previous GWASs have identified six RLS risk loci (*MEIS1, BTBD9, MAP2K5, PTPRD, TOX3*, and an intergenic region on chromosome 2p14) [14–17]; however, only *MEIS1* has been found to be associated with RLS in patients with migraine via candidate gene approach [24]. Hence, susceptibility genes for RLS in migraineurs might not be completely the same as those for RLS in general population. None of the above genes were identified associated with risks of RLS in migraineurs in this study. Whether *CCDC141* and *VSTM2L* also contribute to the risk of RLS in general population remains to be explored.

We have used translational knockdowns (morphants), transcriptional knockdowns, and transient knockouts (crispants) in the zebrafish system to examine the functional relationship of *CCDC141* and *VSTM2L* to the symptoms of RLS and migraine and obtained relatively consistent results. The stable *ccdc141* and *vstm2l* KO lines did not show a decrease in *th*-expressing cells or a hyperkinetic movement in pectoral fin and basically behaved like wildtype embryos. Though unexpectedly, some similar cases have been reported in zebrafish, such as *egfl7* and *slc25a46* [33, 48]. The mechanism of genetic compensation for *egfl7* has been shown to be transcriptional adaptation that is triggered by degradation of the mutated mRNA through nonsense-mediated mRNA decay (NMD) to upregulate sequence-similar genes that thereby enable functional compensation [49, 50]. However, the mechanism for *slc25a46* is currently unknown [48]. The expression of *ccdc141* and *vstm2l* in corresponding KO mutants is decreased (Additional file 12), suggesting a transcriptional adaptation caused by NMD [48, 50]. To overcome the genetic compensation and examine the phenotypes in adult animals, different animal models may help. For example, various mouse *Slc25a46* mutants exhibit a spectrum of disorders similar to those in patients with recessive loss of *SLC25A46* function [51–53].

Our study has several implications. First, although the true biological significance of the genes identified from GWAS for complex disorders is often questioned, our findings provide evidence to support the functional roles of the identified genes which is consistent with the prevailing theories of RLS pathogenesis. Of note, the function of *CCDC141 and VSTM2L* has not been fully elucidated. Further studies for these two genes might provide novel mechanisms of RLS, particularly in patients with migraine. Second, only one previous study had employed zebrafish to evaluate the function of *Meis1* gene; however, the study investigated only hindbrain development [54], without phenotypic studies to simulate RLS. Our study further demonstrated the utility of zebrafish to model the behavioural phenotypes of RLS in humans. Spreading depression (or depolarization) (SD) could be used as a preclinical model for migraine study, particularly migraine with aura [55]. A recent paper has established the method to measure SD in the adult zebrafish tectum [56]; therefore, it can be used to examine the “migraine-like” phenotype in the corresponding adult zebrafish mutants in the future. With accurate diagnoses and strict criteria for the patient recruitment, we obtained significant signals with a limited sample size. However, only common variants were included from the GWAS results in this study. Further investigations are required to look at rare variants with fine mappings. Moreover, we focused on SNPs located in or near a gene in the replication analysis for reasons stated in Methods. The possibility that SNPs not mapped to a gene have roles in pathogenesis remains to be examined. Finally, our findings provide biological insights on the ample clinical evidence supporting the RLS-migraine comorbidity, which may support the implement of a detailed questionnaire about sleep disorder and restless legs symptoms in patients with frequent migraine in clinical practice. For those with symptoms with RLS, testing for iron, ferritin or other secondary causes of RLS may be mandatory. Moreover, it may be appropriate to treat RLS with dopaminergic D2 agonist in patients with migraine, which may be beneficial for both RLS symptoms and migraine in these patients [57].

## Conclusions

To conclude, our study suggests that *CCDC141* and *VSTM2L* are associated with increased risks of RLS in patients with migraine. Interference of these two genes, as explored in zebrafish, leads to RLS-like phenotypes which might be related to dysregulated dopaminergic neurotransmission.

## Supplementary Information

** Additional file 1. **Morpholino sequences. Supplementary **Table 1.** describing morpholino sequencesused in this study.

**Additional file 2. **Primer sequences for sgRNA cloning. Supplementary **Table 2.** including the Primer sequencesfor sgRNA cloning.

**Additional file 3. **Protospacer for making crRNA. Supplementary **Table 3.** detailing the protospacer formaking crRNA.

**Additional file 4. **SNPs selected for association studies for restlesslegs syndrome in patients with migraine. Supplementary **Table 4.** detailing the SNPs selected forassociation studies for restless legs syndrome in patients with migraine.

**Additional file 5. **Association of RLS with SNPs within dopamine receptor or tyrosine hydroxylase genes. Supplementary **Table 5.** detailing the SNPs within genesof dopamine receptor or tyrosine hydroxylase with borderline significantassociation with restless legs syndrome in patients with migraine.

**Additional file 6. **Expression pattern of *ccdc141* and *vstm2l *in 1-4 dpfembryos of zebrafish. Supplementary **Figure 1.** *In situ* hybridization was conducted with *ccdc141* and *vstm2l*antisense RNA probes on wild-type embryos.

**Additional file 7. **Summary of morpholino (MO) results. Supplementary **Table 6.** showing the summary of MOresults.

**Additional file 8. **Hyperkinetic movements of pectoral fins in *vstml2*morphants in comparison with that of wild-type. a video showing that the *vstml2* morphants (right) having a higher fin flappingfrequency than that of wild-type (left).

**Additional file 9. **Summary of CRISPR/dCas9 results. Supplementary **Table 7.** showing the summary of CRISPR/dCas9results.

**Additional file 10. **Gene expression of targeted genes, th expression andfin movement of ccdc141 and vstm2l CRISPRi-injected embryos. Supplementary **Figure 2. **(A) ccdc141 CRISPRi-injected embryos showed (Aa) adecreased gene expression level, (Ab) reduced th-positive amacrine cells and(Ac) hyperkinetic movements compared with non-injected embryos (AB)

**Additional file 11. **Stable KO lines and corresponding genotyping methods. Supplementary **Table 8.** detailing the stable KO linesand corresponding genotyping methods.

**Additional file 12. **The analysis of stable F2 *ccdc141E2* -4 bp and *vstm2l* *E2* -8 bpknocked-out embryos. Supplementary **Fig 3. **The number of *th*-expressing amacrine cells in the homozygousmutants showed no statistically significantdifference, compared with respective sibling controls, including 4 dpf (**A**) *ccdc141* *E2* -4 bp and (**B**) *vstm2l* *E2* -8 bp embryos.

## Data Availability

The details of zebrafish experiments were provided in the Additional files. The other supporting data are available from the corresponding authors upon reasonable request.
