# Supplementary material for: Genome-wide analysis identified novel susceptible genes of restless legs syndrome in migraineurs
Source: J Headache Pain. 2022 Mar 29;23(1):39. doi: 10.1186/s10194-022-01409-9 (PMC8966278; doi:10.1186/s10194-022-01409-9)
Supplement: Supplementary file 1 — Additional file 1. Morpholino sequences. Supplementary Table 1. describing morpholino sequencesused in this study. [file 10194_2022_1409_MOESM1_ESM.docx]

**Supplementary Table 1. Morpholino sequences.**

| **Name** | **Sequence** |
| --- | --- |
| *ccdc141* MO1 | 5’-GTCCACACGTCCAAGAGCAGAAAGA-3’ |
| *ccdc141* MO2 | 5’-GAAAGTCAACGTTACCTCCAAAGCA-3’ |
| *ccdc141* MO3 | 5’-GAGAAACAGAAATCTTACCTCTTCT-3’ |
| *vstm2l* MO1 | 5’- TCTGCTCTACGCTCTCACTAGATCC-3’ |
| *vstm2l* MO2 | 5’-CCTCCCGTTTCTCACACTTACCATT-3’ |
| *vstm2l* MO3 | 5’-ATGCTCTCACTAGATCCTCTGATAA-3’ |
