# Supplementary material for: Genome-wide analysis identified novel susceptible genes of restless legs syndrome in migraineurs
Source: J Headache Pain. 2022 Mar 29;23(1):39. doi: 10.1186/s10194-022-01409-9 (PMC8966278; doi:10.1186/s10194-022-01409-9)
Supplement: Supplementary file 2 — Additional file 2. Primer sequences for sgRNA cloning. Supplementary Table 2. including the Primer sequencesfor sgRNA cloning. [file 10194_2022_1409_MOESM2_ESM.docx]

**Supplementary Table 2. Primer sequences for sgRNA cloning.**

| *ccdc141*_dcas9_gRNA1F: | 5’-TAGGGTTCAGAAGTCCACGACTGA-3’ |
| --- | --- |
| *ccdc141*_dcas9_gRNA1R: | 5’-AAACTCAGTCGTGGACTTCTGAAC-3’ |
| *ccdc141*_dcas9_gRNA2F: | 5’-TAGGGAACAAAGCCCAGTACACAC-3’ |
| *ccdc141*_dcas9_gRNA2R: | 5’-AAACGTGTGTACTGGGCTTTGTTC-3’ |
| *ccdc141*_dcas9_gRNA3F: | 5’-TAGGATCACCTTCACTGGACATTG-3’ |
| *ccdc141*_dcas9_gRNA3R: | 5’-AAACCAATGTCCAGTGAAGGTGAT-3’ |
| *ccdc141*_dcas9_gRNA4F: | 5’-TAGGCACAGTGCTGATGGTTGTTG-3’ |
| *ccdc141*_dcas9_gRNA4R: | 5’-AAACCAACAACCATCAGCACTGTG-3’ |
| *vstm2l*_dcas9_gRNA1F: | 5’-TAGGCTGTGTTCCGCCACACACAC-3’ |
| *vstm2l*_dcas9_gRNA1R: | 5’-AAACGTGTGTGTGGCGGAACACAG-3’ |
| *vstm2l*_dcas9_gRNA2F: | 5’-TAGGAATGGTACCTGTACGTGTGT-3’ |
| *vstm2l*_dcas9_gRNA2R: | 5’-AAACACACACGTACAGGTACCATT-3’ |
| *vstm2l*_dcas9_gRNA3F: | 5’-TAGGCTGCCAGATTCGAGCTGATC-3’ |
| *vstm2l*_dcas9_gRNA3R: | 5’-AAACGATCAGCTCGAATCTGGCAG-3’ |
| *vstm2l*_dcas9_gRNA4F: | 5’-TAGGGGCTCGAGAAAACACATCAG-3’ |
| *vstm2l*_dcas9_gRNA4R: | 5’-AAACCTGATGTGTTTTCTCGAGCC-3’ |
