# Supplementary material for: Genome-wide analysis identified novel susceptible genes of restless legs syndrome in migraineurs
Source: J Headache Pain. 2022 Mar 29;23(1):39. doi: 10.1186/s10194-022-01409-9 (PMC8966278; doi:10.1186/s10194-022-01409-9)
Supplement: Supplementary file 3 — Additional file 3. Protospacer for making crRNA. Supplementary Table 3. detailing the protospacer formaking crRNA. [file 10194_2022_1409_MOESM3_ESM.docx]

**Supplementary Table 3. Protospacer for making crRNA.**

| **Name** | **Sequence** |
| --- | --- |
| *ccdc141* E1 (targeting exon 1) | 5'-CACAGTGCTGATGGTTGTTG-3' |
| *vstm2l* E1 (targeting exon 1) | 5'-GATCAGCTCGAATCTGGCAG-3' |
| *ccdc141* E2 (targeting exon 2) | 5’-GGTGCATATACAGCTGACTG-3’ |
| *vstm2l* E2 (targeting exon 2) | 5’-GTGGTACAGCAGACAATGGG-3’ |
