# Supplementary material for: Genome-wide analysis identified novel susceptible genes of restless legs syndrome in migraineurs
Source: J Headache Pain. 2022 Mar 29;23(1):39. doi: 10.1186/s10194-022-01409-9 (PMC8966278; doi:10.1186/s10194-022-01409-9)
Supplement: Supplementary file 4 — Additional file 4. SNPs selected for association studies for restlesslegs syndrome in patients with migraine. Supplementary Table 4. detailing the SNPs selected forassociation studies for restless legs syndrome in patients with migraine. [file 10194_2022_1409_MOESM4_ESM.docx]

**Supplementary Table 4. SNPs selected for association studies for restless legs syndrome in patients with migraine.**

|  |  | | | | | | | | | | | | **Joint analysis** | | |
| --- | --- | --- | --- | --- | --- | --- | --- | --- | --- | --- | --- | --- | --- | --- | --- |
| **SNP** | | **Gene** | **Chr** | **Position** | **Risk**  **allele** | **stage** | **RAF (RLS)** | **RAF (Control)** | **OR (95%CI)** | | **P value** | **P value** | **OR (95%CI)** | | **P value** |
|  |  |  |  |  |  |  |  |  |  |  | **(Trend)** | **(PC10)** |  |  | **(Trend)** |
| rs6747725 | | *CCDC141* | 2 | 179837049 | G | 2 | 0.9963 | 0.9958 | 1.140 | (0.136-9.551) | 0.904 |  |  |  |  |
| rs79823654 | | *CCDC141* | 2 | 179839018 | A | 1 | 0.130 | 0.053 | 2.740 | (1.715-4.377) | 1.05x10^-5^ | 2.63x10^-5^ |  |  |  |
|  | |  |  |  |  | 2 | 0.101 | 0.061 | 1.642 | (1.084-2.486) | 0.017 |  | 2.041 | (1.499-2.779) | 3.27x10^-6^ |
| rs75704987 | | *CCDC14* | 2 | 179839961 | G | 2 | 1 | 1 |  |  |  |  |  |  |  |
| rs9514634 | | *FAM155A* | 13 | 107885801 | C | 1 | 0.7696 | 0.7555 | 1.077 | (0.778-1.492) | 0.655 | 0.560 |  |  |  |
|  | |  |  |  |  | 2 | 0.8087 | 0.7638 | 1.292 | (0.951-1.754) | 0.1 |  | 1.190 | (0.952-1.487) | 0.1246 |
| rs9520345 | | *FAM155A* | 13 | 107902876 | C | 1 | 0.1913 | 0.1014 | 2.191 | (1.478-3.250) | 6.26 x10^-5^ | 6.56 x10^-5^ |  |  |  |
|  | |  |  |  | T | 2 | 1 | 1 |  |  |  |  |  |  |  |
| rs8114158 | | *VSTM2L* | 20 | 36540794 | A | 1 | 0.237 | 0.115 | 2.277 | (1.609-3.222) | 1.46 x10^-6^ | 2.13x10^-6^ |  |  |  |
|  | |  |  |  |  | 2 | 0.175 | 0.134 | 1.359 | (0.974-1.027) | 0.070 |  | 1.729 | (1.362-2.195) | 5.10x10^-6^ |
| rs6021854 | | *VSTM2L* | 20 | 36545927 | A | 1 | 0.252 | 0.116 | 2.447 | (1.738-3.446) | 8.63 x10^-8^ | 1.86x10^-7^ |  |  |  |
|  | |  |  |  |  | 2 | 0.182 | 0.136 | 1.421 | (1.021-1.977) | 0.036 |  | 1.835 | (1.450-2.324) | 2.73x10^-7^ |

SNPs in blue indicate the significant ones identified in the discovery stage; the other three SNPs were added for fine mapping. In the replication stage, two of the SNPs (rs75704987 in *CCDC14* and rs9520345 in *FAM155A*) failed genotyping and another three (rs6747725 in *CCDC141*, rs9514634 in *FAM155A*, and rs8114158 in *VSTM2L*) did not reach statistical significance.
