# Supplementary material for: Genome-wide analysis identified novel susceptible genes of restless legs syndrome in migraineurs
Source: J Headache Pain. 2022 Mar 29;23(1):39. doi: 10.1186/s10194-022-01409-9 (PMC8966278; doi:10.1186/s10194-022-01409-9)
Supplement: Supplementary file 5 — Additional file 5. Association of RLS with SNPs within dopamine receptor or tyrosine hydroxylase genes. Supplementary Table 5. detailing the SNPs within genesof dopamine receptor or tyrosine hydroxylase with borderline significantassociation with restless legs syndrome in patients with migraine. [file 10194_2022_1409_MOESM5_ESM.docx]

Supplementary Table 5. Association of RLS with SNPs within dopamine receptor or tyrosine hydroxylase genes

| SNP | Gene | Chr | Position | Risk allele | Risk allele frequency | | OR | P value (Trend) | P value (adjusted) |
| --- | --- | --- | --- | --- | --- | --- | --- | --- | --- |
|  |  |  |  |  | Case | Control |  |  |  |
| rs2434245 | *DRD1* | 5 | 175207199 | A | 0.174 | 0.094 | 2.062 | 2.7×10^-4^ | 3.2×10^-4^ |
| rs2434244 | *DRD1* | 5 | 175208535 | A | 0.174 | 0.094 | 2.062 | 2.7×10^-4^ | 3.2×10^-4^ |
| rs2434243 | *DRD1* | 5 | 175208548 | T | 0.174 | 0.093 | 2.079 | 2.2×10^-4^ | 2.7×10^-4^ |
| rs2122898 | *DRD1* | 5 | 175208758 | C | 0.178 | 0.094 | 2.112 | 1.5×10^-4^ | 1.9×10^-4^ |
| rs2443543 | *DRD1* | 5 | 175222418 | T | 0.170 | 0.091 | 2.046 | 3.4×10^-4^ | 3.6×10^-4^ |
| rs11822504 | *TH* | 11 | 1663950 | C | 0.296 | 0.421 | 0.583 | 4.4×10^-4^ | 5×10^-4^ |
| rs2334489 | *TH* | 11 | 1665733 | G | 0.113 | 0.217 | 0.460 | 3.1×10^-4^ | 3.1×10^-4^ |
| rs59724260 | *TH* | 11 | 1676757 | T | 0.113 | 0.225 | 0.442 | 1.4×10^-4^ | 1.6×10^-4^ |
| rs61867601 | *TH* | 11 | 1677131 | A | 0.113 | 0.222 | 0.452 | 2×10^-4^ | 2.4×10^-4^ |
| rs11825487 | *TH* | 11 | 1679397 | T | 0.091 | 0.186 | 0.435 | 4.4×10^-4^ | 4.7×10^-4^ |
| rs7118687 | *DRD2* | 11 | 113518429 | C | 0.517 | 0.391 | 1.713 | 2.5×10^-4^ | 1.6×10^-4^ |
| rs7110543 | *DRD2* | 11 | 113524040 | G | 0.517 | 0.394 | 1.694 | 3.6×10^-4^ | 2.3×10^-4^ |
| rs200368603 | *DRD2* | 11 | 113528990 | G | 0.517 | 0.394 | 1.698 | 3.4×10^-4^ | 2.1×10^-4^ |
| rs10891583 | *DRD2* | 11 | 113535757 | G | 0.517 | 0.394 | 1.698 | 3.4×10^-4^ | 2.1×10^-4^ |
| rs12421282 | *DRD2* | 11 | 113537984 | T | 0.517 | 0.394 | 1.698 | 3.4×10^-4^ | 2.1×10^-4^ |
| rs4938034 | *DRD2* | 11 | 113548921 | A | 0.513 | 0.391 | 1.695 | 3.7×10^-4^ | 2.4×10^-4^ |
| rs7116612 | *DRD2* | 11 | 113552234 | A | 0.513 | 0.392 | 1.677 | 4.6×10^-4^ | 3.1×10^-4^ |

Abbreviations: SNP, single nucleotide polymorphism; Chr, chromosome; OR, odds ratio for risk allele;

P value is derived from trend test, the P value adjusted is derived from the logistic regression adjusted with age and sex; Risk allele, allele with higher frequency in cases compared to controls. All genomic information is from human genome build hg19.
