# Supplementary figures and images for: Genome-wide analysis identified novel susceptible genes of restless legs syndrome in migraineurs
Source: J Headache Pain. 2022 Mar 29;23(1):39. doi: 10.1186/s10194-022-01409-9 (PMC8966278; doi:10.1186/s10194-022-01409-9)

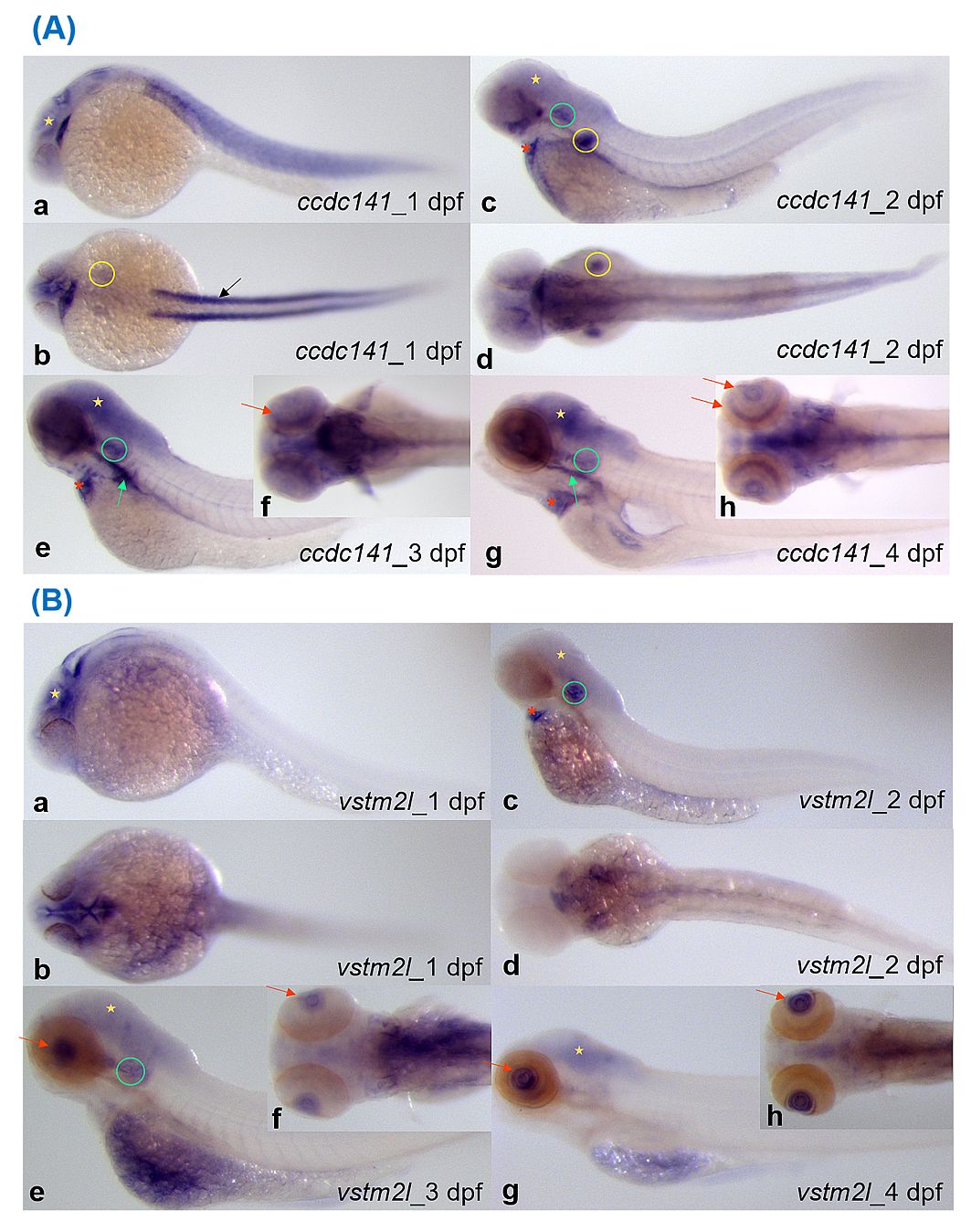

Supplement: Supplementary file 6 — Additional file 6. Expression pattern of ccdc141 and vstm2l in 1-4 dpfembryos of zebrafish. Supplementary Figure 1. In situ hybridization was conducted with ccdc141 and vstm2lantisense RNA probes on wild-type embryos. [file 10194_2022_1409_MOESM6_ESM.jpg]

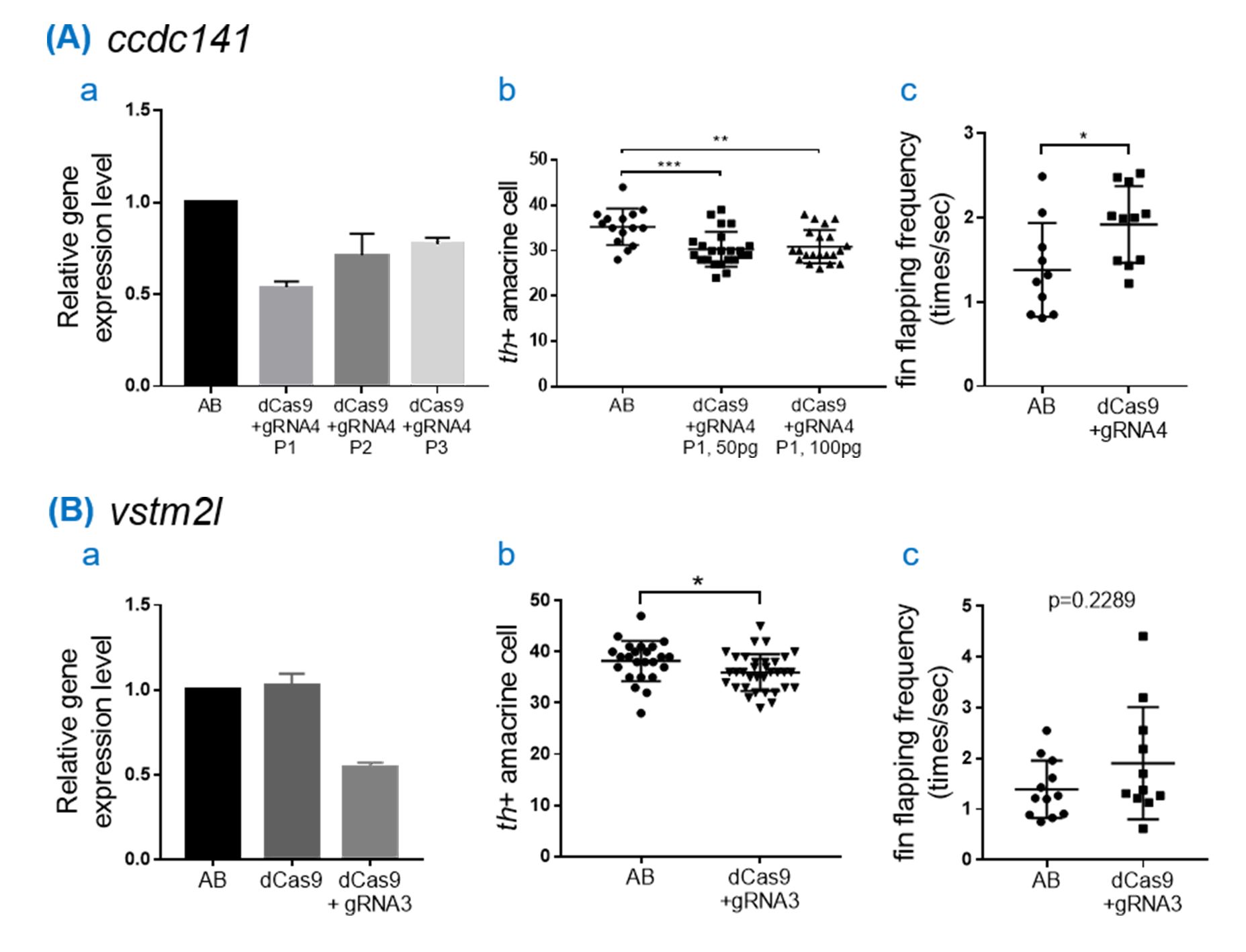

Supplement: Supplementary file 10 — Additional file 10. Gene expression of targeted genes, th expression andfin movement of ccdc141 and vstm2l CRISPRi-injected embryos. Supplementary Figure 2. (A) ccdc141 CRISPRi-injected embryos showed (Aa) adecreased gene expression level, (Ab) reduced th-positive amacrine cells and(Ac) hyperkinetic movements compared with non-injected embryos (AB) [file 10194_2022_1409_MOESM10_ESM.jpg]

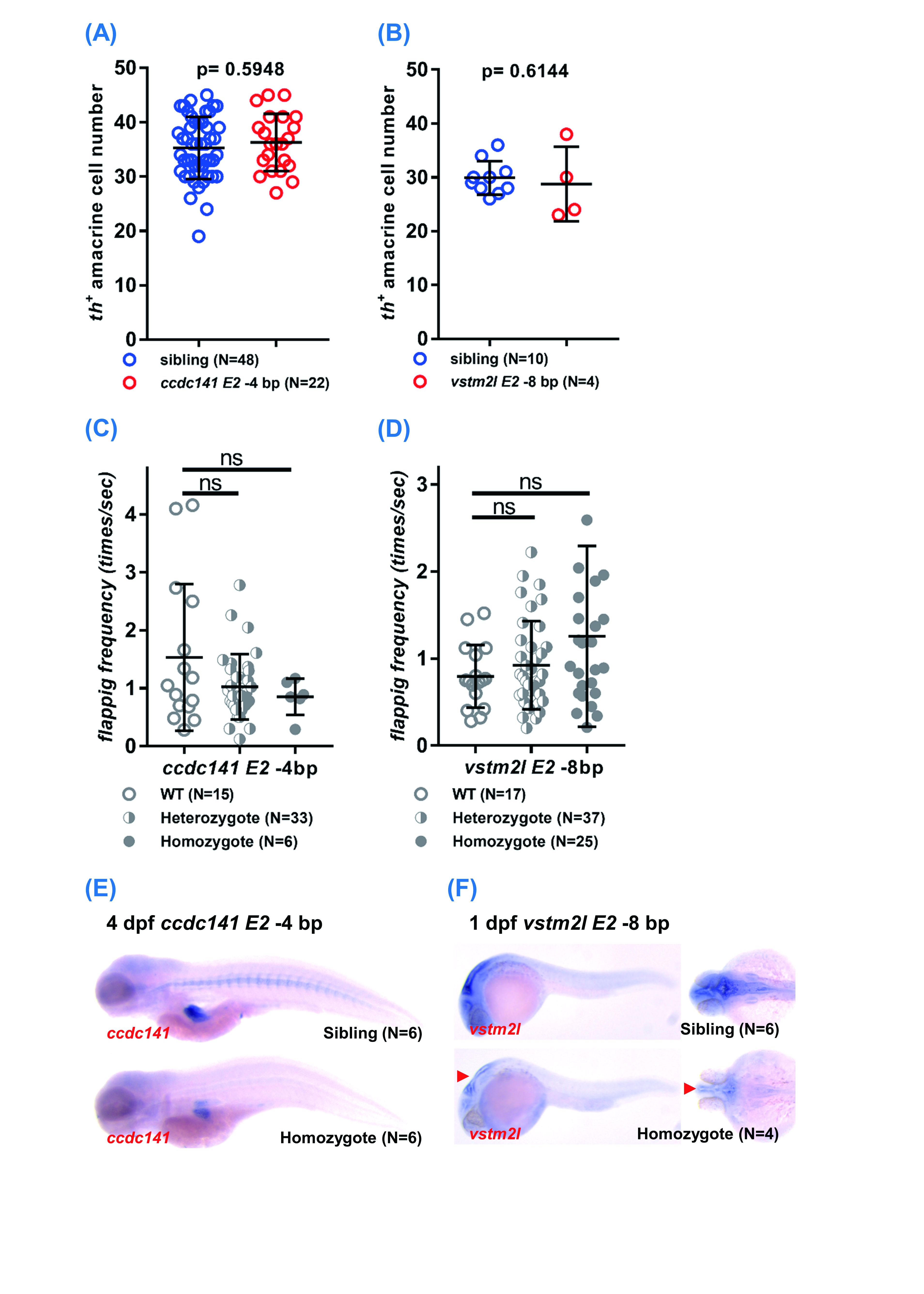

Supplement: Supplementary file 12 — Additional file 12. The analysis of stable F2 ccdc141E2 -4 bp and vstm2l E2 -8 bpknocked-out embryos. Supplementary Fig 3. The number of th-expressing amacrine cells in the homozygousmutants showed no statistically significantdifference, compared with respective sibling controls, including 4 dpf (A) ccdc141 E2 -4 bp and (B) vstm2l E2 -8 bp embryos. [file 10194_2022_1409_MOESM12_ESM.jpg]
