# Supplementary material for: Genome-wide analysis identified novel susceptible genes of restless legs syndrome in migraineurs
Source: J Headache Pain. 2022 Mar 29;23(1):39. doi: 10.1186/s10194-022-01409-9 (PMC8966278; doi:10.1186/s10194-022-01409-9)
Supplement: Supplementary file 7 — Additional file 7. Summary of morpholino (MO) results. Supplementary Table 6. showing the summary of MOresults. [file 10194_2022_1409_MOESM7_ESM.docx]

**Supplementary Table 6. Summary of MO results.**

| Morpholino | Phenotype observation | In situ (dopaminergic cell) | Fin observation | Gene Rescue | Morpholino Effect |
| --- | --- | --- | --- | --- | --- |
| *ccdc141* MO1 | growth-retarded  Edema  Fin deformity | amacrine cells decreased^a^ | no difference (P=0.5617)^b^ | recovery the amacrine cell number^c^ | - |
| *ccdc141* MO2 | no obvious phenotype | no difference | no difference | - | in-frame deletion |
| *ccdc141* MO3 | curly tail down | - | - | - | no splicing blocking effect |
| *vstm2l* MO1 | curly tail/edema/  gastrointestinal bleeding | no difference | no difference | - | - |
| *vstm2l* MO2 | edema/  gastrointestinal bleeding | pretectum, superior cervical ganglion (SCG), amacrine cells decreased^d^ | hyperkinetic movements (P=0.0159)^e^ | - | pre-terminated |
| *vstm2l* MO3 | gastrointestinal bleeding | amacrine cells decreased | no difference | - | - |

^a^See Figure 3D; ^b^See Figure 3H; ^c^See Figure 3F; ^d^See Figure 3E; ^e^See Figure 3G.
