# Supplementary material for: Genome-wide analysis identified novel susceptible genes of restless legs syndrome in migraineurs
Source: J Headache Pain. 2022 Mar 29;23(1):39. doi: 10.1186/s10194-022-01409-9 (PMC8966278; doi:10.1186/s10194-022-01409-9)
Supplement: Supplementary file 9 — Additional file 9. Summary of CRISPR/dCas9 results. Supplementary Table 7. showing the summary of CRISPR/dCas9results. [file 10194_2022_1409_MOESM9_ESM.docx]

**Supplementary Table 7. Summary of CRISPR/dCas9 results.**

|  | Phenotype observation | In situ (dopaminergic cell) | Fin observation | qRT-PCR |
| --- | --- | --- | --- | --- |
| *ccdc141* gRNA1 | pericardial & yolk edema  notochord bend  Cyclopia | amacrine cells decreased  (P=0.0101) | no difference  (P=0.3474, 0.3631) | *ccdc141* expression decreased  (0.434-fold) |
| *ccdc141* gRNA2 | pericardial & yolk edema  notochord bend  Cyclopia | no difference  (P=0.2496) | - | no repression |
| *ccdc141* gRNA3 | pericardial & yolk edema  notochord bend  Cyclopia | no difference  (P=0.5027, 0.0858) | no difference | *ccdc141* expression decreased  (0.548-fold) |
| *ccdc141* gRNA4 | pericardial & yolk edema  notochord bend  Cyclopia | amacrine cells decreased  (100 pg, P=0.0024^a^, 50 pg, P=0.0010^b^) | hyperkinetic movements^c^ | *ccdc141* expression decreased  (0.538-fold) |
| *vstm2l* gRNA1 | pericardial & yolk edema  notochord bend  Cyclopia | - | - | no repression |
| *vstm2l* gRNA2 | pericardial & yolk edema  notochord bend  Cyclopia | - | - | no repression |
| *vstm2l* gRNA3 | pericardial & yolk edema  notochord bend  Cyclopia | amacrine cells decreased  (100 pg, P=0.0157^d^) | no difference  (100 pg, P=0.3582, 0.2289^e^) | *vstm2l* expression decreased  (50 pg=0.54-fold) |
| *vstm2l* gRNA4 | pericardial & yolk edema  notochord bend  Cyclopia | no difference  (P=0.1334) | - | 50 pg= 0.73-fold  100 pg= 0.9-fold |

^a^See Figure S2Ab, 100 pg; ^b^See Figure S2Ab, 50 pg; ^c^See Figure S2Ac; ^d^See Figure S2Bb; ^e^See Figure S2Bc.
