# Supplementary material for: Genome-wide analysis identified novel susceptible genes of restless legs syndrome in migraineurs
Source: J Headache Pain. 2022 Mar 29;23(1):39. doi: 10.1186/s10194-022-01409-9 (PMC8966278; doi:10.1186/s10194-022-01409-9)
Supplement: Supplementary file 11 — Additional file 11. Stable KO lines and corresponding genotyping methods. Supplementary Table 8. detailing the stable KO linesand corresponding genotyping methods. [file 10194_2022_1409_MOESM11_ESM.docx]

**Supplementary Table 8. Stable KO lines and corresponding genotyping methods.**

| **Allele** | **Genomic sequencing results** | **predicted protein** | **Genotyping primers** | **Restriction enzyme and result** |
| --- | --- | --- | --- | --- |
| *ccdc141* E2 (WT) | TGGATACAAGAGATGCATGTACAGTAGCACAGCCATACATTAACACCTCTCACTCTCACTCTCTGTATGTGTGTGTTTTAAAGTGTGGGGAGCTGGTGCATATACAGCTGACTGAGGCACAGCCTAACCTCCTGGAGATTGGAAAC | **WT Ccdc141** |  |  |
| *ccdc141* E2 -10 bp | TGGATACAAGAGATGCATGTACAGTAGCACAGCCATACATTAACACCTCTCACTCTCACTCTGTGTATGTGTGTGTTTTAAAGTGTGGGGAGCTGGTGCATATXXXXXXXXXXGAGGCACAGCCTAACCTCCTGGAGATTGGAAAC | **p.Ile41Metfs*38** |  |  |
| *ccdc141* E2 -4 bp | TGGATACAAGAGATGCATGTACAGTAGCACAGCCATACATTAACACCTCTCACTCTCACTCTGTGTATGTGTGTGTTTTAAAGTGTGGGGAGCTGGTGCATATACAGCTGAXXXXGGCACACCCTAACCTCCTGGAGATTGGAAAC | **p.Thr44Argfs*37** | 5’GGACTTGCAATAGATCGGAT3’  5’AATAAAGCCCTGACCTGAAG3’ | BbvCI cuts mutant amplicon |
| *ccdc141* E2 +1 bp | TGGATACAAGAGATGCATGTACAGTAGCACAGCCATACATTAACACCTCTCACTCTCACTCTGTGTATGTGTGTGTTTTAAAGTGTGGGGAGCTGGTGCATATACAGCTGAACTGAGGCACAGCCTAACCTCCTGGAGATTGGAAAC | **p.Thr44Lysfs*1** |  |  |
| *vstm2l*  E2 (WT) | TTCCTCGTCCGTCTCGCTGGAGATCCAGTGGTGGTACAGCAGACAATGGGCGGAGCCATTGCCGTGGGCAACCAATCAGGAAAGACACACTTTCGCATAATCATTTGTTCTTTACCTTATGCAAATTGTTTGGTGCAGTTTTACCAGAAAAGGTTTTCGCATTTTTAAT | **WT Vstm2l** |  |  |
| *vstm2l*  E2 -8 bp | TTCCTCGTCCGTCTCGCTGGAGATCCAGTGGTGGTACAGCAGACAXXXXXXXXAGCCATTGCCGTGGGCAACCAATCAGGAAAGACACACTTTCGCATAATCATTTGTTCTTTACCTTATGCAAATTGTTTGGTGCAGTTTTACCAGAAAAGGTTTTCGCATTTTTAAT | **p.Trp77Alafs*20** | 5’TCGAGCCGGAGAAGATGTG3’  5’CCTTTTCTGGTAAAACTGCACC3’ | EciI cuts WT amplicon |
| *vstm2l*  E2 -13 bp | TTCCTCGTCCGTCTCGCTGGAGATCCAGTGGTGGTACAGCAGXXXXXXXXXXXXXCCATTGCCGTGGGCAACCAATCAGGAAAGACACACTTTCGCATAATCATTTGTTCTTTACCTTATGCAAATTGTTTGGTGCAGTTTTACCAGAAAAGGTTTTCGCATTTTTAAT | **p.Arg75Serfs*25** |  |  |

Note: Letter X means deleted nucleotide; letter in blue means inserted nucleotide.
